# Prognostic lncRNA, miRNA, and mRNA Signatures in Papillary Thyroid Carcinoma

**DOI:** 10.3389/fgene.2020.00805

**Published:** 2020-08-04

**Authors:** Kun Wang, Jing Xu, Lu Zhao, Shiyang Liu, Chenguang Liu, Lin Zhang

**Affiliations:** Department of Thyroid and Breast Surgery, Tongji Hospital, Tongji Medical College, Huazhong University of Science and Technology, Wuhan, China

**Keywords:** papillary thyroid carcinoma, long non-coding RNA, microRNA, messenger RNA, nomogram, prognosis

## Abstract

The current focus in the treatment of papillary thyroid carcinoma (PTC) is tumor progression. The aim of this study was to build RNA-based classifiers and develop a comprehensive model to provide progression-free interval (PFI) risk prediction for PTC. The RNAseq data, miRNAseq data, and clinical information of PTC were downloaded from The Cancer Genome Atlas database. Based on the differently expressed RNAs, the least absolute shrinkage and selection operator (LASSO) Cox regression model was utilized to build the RNA-based classifiers for PFI of the patients with PTC. A 6-messenger RNA (mRNA)-based classifier, a 5-long non-coding RNA (lncRNA)-based classifier, and a 4-microRNA (miRNA)-based classifier were constructed to predict the PFI. Patients with high risk based on the constructed RNA-based classifiers had worse prognosis in Kaplan–Meier curve analysis with log-rank test. The areas under the curves of the first, third, and fifth years in the training and testing set were 0.83, 0.82, and 0.82 and 0.67, 0.72, and 0.73 for the 6-mRNA-based classifier, respectively; 0.75, 0.84, and 0.85 and 0.71, 0.67, and 0.71 for the 5-lncRNA-based classifier, respectively; and 0.70, 0.77, and 0.79 and 0.74, 0.67, and 0.66 for the 4-miRNA-based classifier, respectively. The prediction capability of the three RNA-based classifiers was superior to the TNM stage system. Furthermore, a nomogram based on the verified independent prognostic factors was established for the prognostic prediction. The C-index and calibration plots indicated good predictive accuracy of the nomogram. In summary, the 6-mRNA-based classifier and 5-lncRNA-based classifier constructed in this study were independent prognostic factors for PTC.

## Introduction

Thyroid carcinoma (THCA) is the most prevalent malignancy of the endocrine system. The incidence rate of THCA nearly tripled between 1975 and 2016 (Howlader et al., 2019). Papillary thyroid carcinoma (PTC), the most common and least deadly histologic type, accounts for up to 90% of the new cases (Howlader et al., 2019). Although the morbidity rate of PTC has risen, the mortality from the cancer has been relatively stable (Howlader et al., 2019). PTC tends to have a biologically indolent nature and can be effectively treated with surgical operation, hormone therapy, and radioiodine therapy. The 30-year cancer-specific death rate from PTC was less than 10% (Grogan et al., 2013). However, the 30-year recurrence rate was close to 30% (Dong et al., 2019).

Patients suffering PTC progression would live under enormous mental stress and financial burden, although most of the progressions were not lethal. Thus, more clinical attention should be paid to PTC progression. It is crucial to explore the molecular mechanisms underlying PTC progression. Several molecular changes have been identified in PTC. RET fusion proteins (the RET/PTC family) appeared to play an oncogenic role in approximately 20% of PTC (Prescott and Zeiger, 2015). Mutation in the BRAF gene resulting in the BRAF V60E protein was also considered prominent in PTC. Many studies focused on molecular markers in predicting outcome for patients with PTC and found some widely known molecular markers, such as BRAF, RAS, RET, and TERT. However, the majority of studies were retrospective and large prospective studies are needed (D’Cruz et al., 2018).

Long non-coding RNAs (lncRNAs), defined as RNA molecules with longer than 200 nucleotides and without translation function, have gained widespread attention in the last few years as a potentially new and essential layer of multiple biological processes. Competing endogenous RNA (ceRNA) network was proposed as a specific regulatory pathway of lncRNAs, by which lncRNAs could sponge microRNAs (miRNAs) through miRNA response elements and participate in the regulation of target RNA expression subsequently (Salmena et al., 2011; Thomson and Dinger, 2016). LncRNAs have been suggested as crucial factors in oncogenesis and tumor development (Tsai et al., 2011; Schmitt and Chang, 2016). Some researchers have revealed that several lncRNAs were associated with the initiation and progression of PTC (Ding et al., 2018; Xia et al., 2018; Zhuang et al., 2019). The ceRNA network has also been investigated in the study about the roles of lncRNAs in the diagnosis and overall survival of PTC (You et al., 2018; Zhao et al., 2018). However, the role of ceRNA network and the prognostic value of lncRNAs, miRNAs, and messenger RNAs (mRNAs) in PTC progression have not been fully explored yet.

In the present study, we performed a comprehensive analysis of PTC progression to identify prognostic lncRNAs, miRNAs, and mRNAs, construct a progression-free interval (PFI)-related ceRNA network, and further develop a nomogram to predict progression risk for PTC patients.

## Materials and Methods

### Data Source

The RNA sequencing data including lncRNAs, miRNAs, and mRNAs of PTC were downloaded from The Cancer Genome Atlas (TCGA) dataset^1^. There were 510 thyroid cancer tissues and 58 adjacent non-tumor tissues for lncRNAs and mRNAs and 514 thyroid cancer tissues and 59 adjacent non-tumor tissues for miRNAs. The corresponding clinical information was downloaded from the UCSC Xena website^2^ and TCGA Pan-Cancer Clinical Data Resource (Liu J. et al., 2018).

### Data Processing

Gene expression quantification data were annotated by Ensembl GTF file in our study. We used edgeR package of R software to identify the differentially expressed lncRNAs (DElncRNAs), mRNAs (DEmRNAs) and miRNAs (DEmiRNAs) with |logFC| >1 and padj <0.05 between PTC and normal tissues. Only the RNAs with a cpm greater than 1 in 2 or more samples were kept in the differential analysis. Patients with complete information on PFI were randomly split into training and testing sets at a 1:1 ratio. In the training set, we first conducted a univariate Cox regression to assess the associations between PFI and the DElncRNAs, DEmiRNAs, and DEmRNAs. RNAs with a *P* < 0.05 in the univariate Cox regression analysis were retained for further analysis. Then, we utilized the least absolute shrinkage and selection operator (LASSO) method with 10-fold cross validation and Cox proportional hazards model with Akaike information criterion (AIC) selection criteria to build the RNA-based classifier. The prognostic accuracy of the classifiers in the training and testing sets was evaluated by the Kaplan–Meier curve, log-rank test, and receiver operating characteristic (ROC) curve analysis. Subsequently, univariate and multivariate Cox regression analysis were performed among the patients with complete RNA-based classifiers and clinical data to select independent prognostic factors. The final model selection was performed by a backward stepwise process with the AIC. A nomogram was further constructed with the final model to estimate the PFI for PTC patients. For nomogram validation, the discrimination was measured via concordance index (c-index) and the calibration was assessed by calibration plots.

### Competing Endogenous RNA Network Construction

The ceRNA network was established based on lncRNA–miRNA–mRNA axes. We used miRcode^3^ database to predict the lncRNA–miRNA interactions. The miRNA–mRNA interactions were identified by the intersection of TargetScan^4^, miRTarBase^5^, and miRDB^6^ database. First, we constructed a global ceRNA network based on the DElncRNAs, DEmiRNAs, and DEmRNAs. Subsequently, we used the PFI-related lncRNAs and mRNAs that were identified by the univariate Cox regression to retrieve lncRNA–miRNA–mRNA axes from the global ceRNA network and form the PFI-related ceRNA network. Cytoscape version 3.7.2 was used to visualize the ceRNA network (Shannon et al., 2003).

### Statistical Analysis

Kaplan–Meier method and log-rank test were used to assess differences between survival curves. LASSO selection method and Cox proportional hazards regression were fitted to the survival data. Chi-square test was used for association analyses. All the analyses were performed in R software (version 3.6.1) with the following packages: “edgeR,” “survival,” “glmnet,” “survminer,” “survivalROC,” and “rms.”

## Results

### Selection of RNAs Related to Progression-Free Interval of Papillary Thyroid Carcinoma and Progression-Free Interval-Related Competing Endogenous RNA Network Construction

**FIGURE 1:**
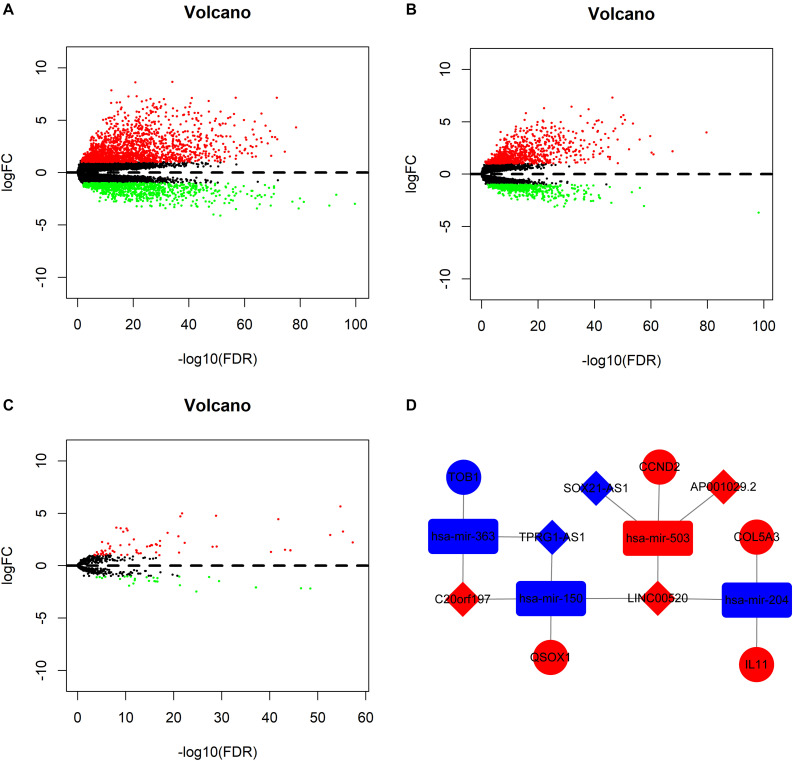
Volcano plot of differentially expressed messenger RNAs (mRNAs) **(A)**, long non-coding RNAs (lncRNAs) **(B)**, and microRNAs (miRNAs) **(C)**. **(D)** Progression-free interval-related competing endogenous RNA network. The red indicates of the upregulated RNAs, and the blue indicates the downregulated RNAs. The diamond represents lncRNAs, the round rectangle represents miRNAs, and the ellipse represents mRNAs.

Nine non-PTC tumor tissues were removed before differential expression analysis. With the cutoff values of |logFC| >1 and *P* <0.05, the edgeR package was utilized to assess the differential expression of RNAs. In total, 2,720 mRNAs (1,750 upregulated and 970 downregulated mRNAs) and 1,330 lncRNAs (848 upregulated and 482 downregulated lncRNAs) were identified between 501 PTC and 58 normal samples (Figures 1A,B), and 91 miRNAs (63 upregulated and 28 downregulated miRNAs) were identified between 505 PTC and 59 normal samples (Figure 1C). From the differently expressed RNAs, we further identified 123 PFI-related lncRNAs, 209 PFI-related mRNAs, and nine PFI-related miRNAs using the univariate Cox regression analysis.

The global ceRNA network built based on DEmRNAs, DEmiRNAs, and DElncRNAs was comprised of 108 lncRNAs, 105 miRNAs, 78 mRNAs, and 460 edges (Supplementary Figure S1). A total of five lncRNAs, four miRNAs, five mRNAs, and 14 edges were retrieved from the global ceRNA network to form the PFI-related ceRNA network (Figure 1D).

### Construction of RNA-Based Classifiers for Progression-Free Interval

**FIGURE 2:**
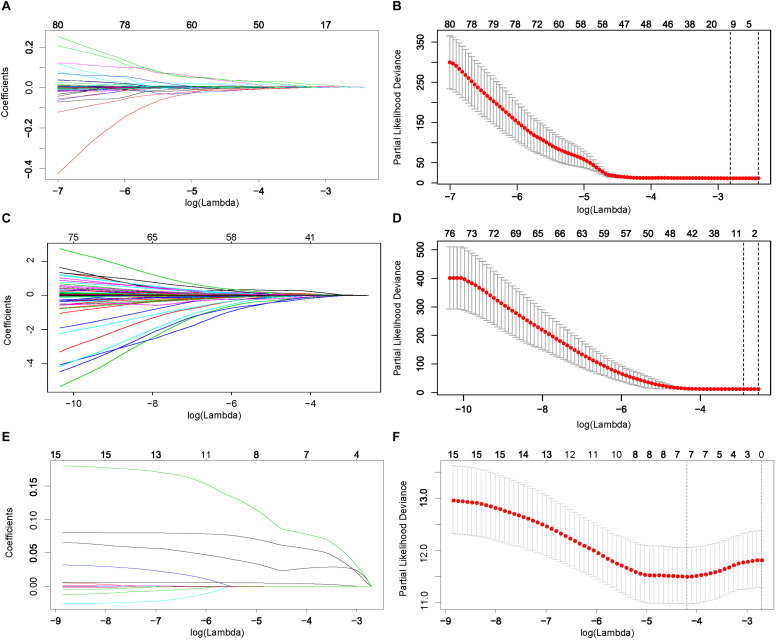
Least absolute shrinkage and selection operator (LASSO) coefficient profiles **(A)** and 10-fold cross validation for tuning parameter selection **(B)** for messenger RNAs (mRNAs). Similarly, **(C,D)** are for long non-coding RNAs (lncRNAs), and **(E,F)** are for microRNAs (miRNAs).

Patients with complete information on PFI were randomly split into training and testing sets at a 1:1 ratio. The distribution of the clinical variables in the training and testing sets was shown in Supplementary Table S1. Based on the PFI-related RNAs identified in univariate Cox regression analysis, we constructed a 6-mRNA-based classifier (Figures 2A,B), a 5-lncRNA-based classifier (Figures 2C,D), and a 4-miRNA-based classifier (Figures 2E,F) for PFI of PTC patients by using the LASSO Cox regression method and AIC selection criteria in the training sets. Details of these lncRNAs, mRNAs, and miRNAs are presented in Table 1.

**TABLE 1:** The details of RNAs for constructing the prognostic signature.

Gene name	ENSG_ID	Chromosome	HR	HR.95L	HR.95H
**6-mRNA-based classifier**					
PIMREG	ENSG00000129195	17p13.2	1.0091	1.0039	1.0143
PAQR4	ENSG00000162073	16p13.3	1.0017	1.0006	1.0029
RHBDL1	ENSG00000103269	16p13.3	1.002	1.0008	1.0032
JAKMIP3	ENSG00000188385	10q26.3	1.0153	1.0085	1.0222
GAS2L2	ENSG00000270765	17q12	1.0038	1.0002	1.0076
PLEKHG4	ENSG00000196155	16q22.1	1.0003	1.0001	1.0005

**5-lncRNA-based classifier**					

AC136475.1	ENSG00000251661	11p15.5	0.9547	0.914	0.9971
LINC02154	ENSG00000235385	Xp22.2	1.033	1.0199	1.0463
AC005082.1	ENSG00000226816	7p15.3	0.9903	0.983	0.9976
AC010969.1	ENSG00000188525	2p25.1	1.094	1.0327	1.1589
AC064805.2	ENSG00000264659	17q25.1	1.0418	1.0205	1.0636

**4-miRNA-based classifier**					

hsa-mir-486-1	ENSG00000274705	8p11.21	0.9987	0.9973	1.0001
hsa-mir-6854	ENSG00000278412	9q22.33	1.0805	1.0155	1.1496
hsa-mir-513c	ENSG00000216171	Xq27.3	1.1247	1.0452	1.2101
hsa-mir-96	ENSG00000199158	7q32.2	1.0055	1.0008	1.0103

**FIGURE 3:**
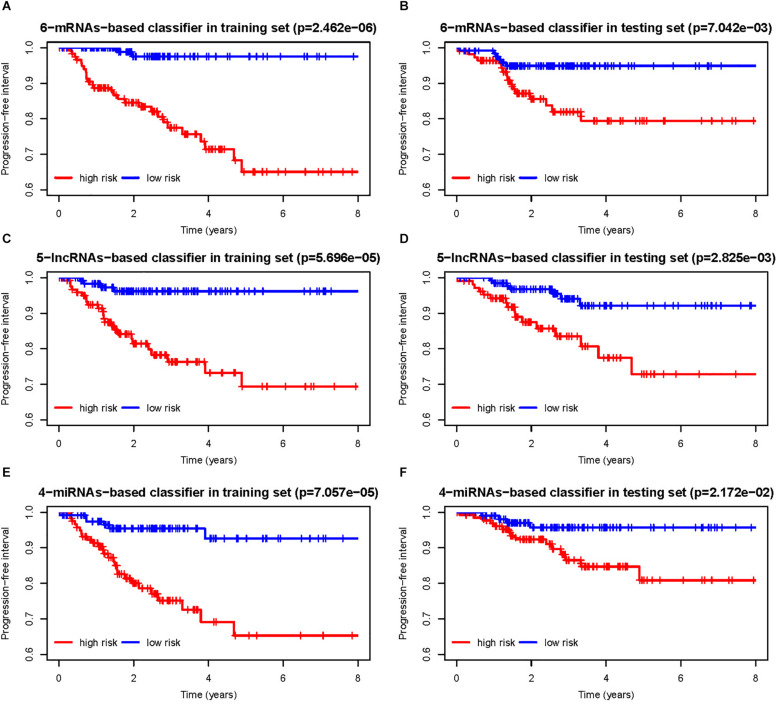
**(A,B)** Progression-free interval curves of papillary thyroid carcinoma (PTC) patients in the training and testing sets with a low or high risk of progression according to 6-messenger RNA (mRNA)-based classifier. Similarly, **(C,D)** are for 5-long non-coding RNA (lncRNA)-based classifier, and **(E,F)** are for 4-microRNA (miRNA)-based classifier.

According to the risk score calculated by the RNA-based classifiers for each patient, patients were divided into high- and low-risk groups using the cutoff of the median risk score (Supplementary Figure S2). The Kaplan–Meier curves and log-rank test showed that patients with high-risk scores had worse PFI than those with low-risk scores in both training cohorts (Figures 3A,C,E) and the testing cohorts (Figures 3B,D,F) for all three classifiers.

As shown in Table 2, for all the three RNA-based classifiers, the pathologic stage showed significant differences between the low-risk and high-risk group in the whole cohort. Patients with a high pathologic T stage were inclined to have a high-risk score, although statistic differences were only marginal for the 5-lncRNA-based and 6-mRNA-based classifiers. Patients with a pathologic subtype of tall cell tended to have a high-risk score according to 6-mRNA-based classifier and 4-miRNAs-based classifier. Older (no less than 55 years) patients might have a high-risk score of 6-mRNA-based classifier. However, gender and pathologic N stage were not associated with risk score of all the three classifiers.

**TABLE 2:** Relationships between risk score of the RNA-based classifier and clinical parameters.

Parameters	Risk of 6-mRNA-based classifier			Risk of 5-lncRNA-based classifier			Risk of 4-miRNA-based classifier		
	Low risk	High risk	p	Low risk	High risk	p	Low risk	High risk	p
Age			0.049			0.14			0.122
<55 years	140	131		151	120		146	125	
≥55 years	55	80		64	71		61	74	
Gender			0.99			0.106			0.076
Female	153	146		166	133		152	147	
Male	54	53		49	58		43	64	
Focus type			0			0.297			0.119
Unifocal	90	121		106	105		93	118	
Multifocal	117	78		109	86		102	93	
Pathologic stage			0.05			0.012			0
Stage I/II	142	121		151	112		146	117	
Stage III	47	45		46	46		32	60	
Stage IV	18	33		18	33		17	34	
Pathologic T stage			0.059			0.078			0.011
T1/2	137	112		142	107		133	116	
T3	64	74		66	72		57	81	
T4	6	13		7	12		5	14	
Pathologic N stage			0.33			1			0.067
N0	114	99		113	100		112	101	
N1	93	100		102	91		83	110	
Pathologic subtype			0.046			0.124			0.022
Classical	158	142		165	135		145	155	
Follicular	39	34		38	35		41	32	
Tall cell	10	23		12	21		9	24	

### Prognostic Value of RNA Classifiers for Assessing Progression-Free Interval

**FIGURE 4:**
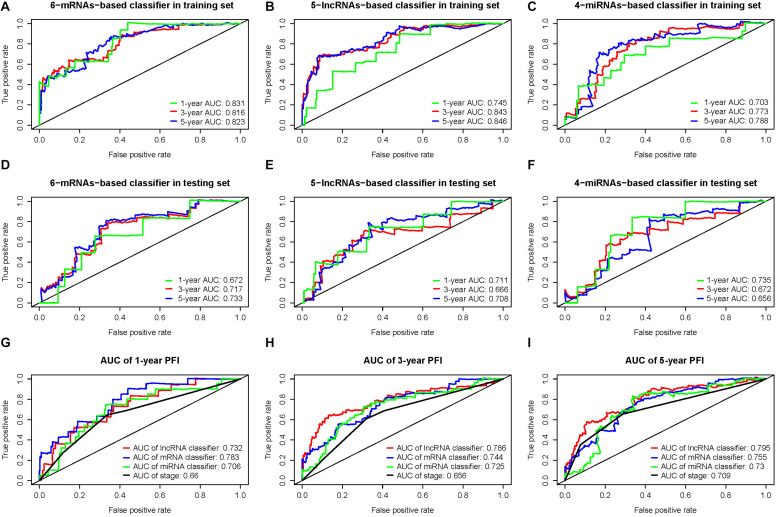
**(A,D)** Time-dependent receiver operating characteristic (ROC) curves at 1, 3, and 5 years for 6-messenger RNA (mRNA)-based classifier in the training and testing sets. Similarly, **(B,E)** are for 5-long non-coding RNA (lncRNA)-based classifier, and **(C,F)** are for 4-microRNA (miRNA)-based classifier. **(G–I)** The ROC for the three RNA classifiers and TNM stage system at 1, 3, and 5 years.

The time-dependent ROC curve analysis revealed that all three classifiers showed a predictive capability. The areas under the curve (AUCs) of the first, third, and fifth years in the training and testing sets were 0.83, 0.82, and 0.82 and 0.67, 0.72, and 0.73, respectively, for the 6-mRNA-based classifier (Figures 4A,D); 0.75, 0.84, and 0.85 and 0.71, 0.67, and 0.71, respectively, for the 5-lncRNA-based classifier (Figures 4B,E); 0.70, 0.77, and 0.79 and 0.74, 0.67, and 0.66, respectively, for the 4-miRNA-based classifier (Figures 4C,F). The ROC curve analysis comparing pathologic TNM stage with the three RNA-based classifiers showed that each of the RNA-based classifiers had better predictive accuracy (Figures 4G–I).

Complete clinical information and RNA-based classifier data were available for further analysis in 406 PTC patients. In the univariate Cox regression analysis, the 6-mRNA-based classifier, 5-lncRNA-based classifier, 4-miRNA-based classifier, age, pathologic stage, pathologic T stage, and pathologic subtype were significantly associated with PFI. However, only the 6-mRNA-based classifier, 5-lncRNA-based classifier, and pathologic T stage were retained to be independent prognostic factors for PFI after the multivariate Cox regression analysis with AIC selection criteria (Table 3).

**TABLE 3:** Univariate and multivariate Cox regression analysis of the three RNA-based classifiers and clinical parameters with progression-free interval.

Parameters	Univariate Cox		Final model	
	HR (95% CI)	*P*	HR (95% CI)	*P*
Risk score of 6-mRNA-based classifier	1.032 (1.022, 1.042)	<0.001	1.030 (1.018, 1.042)	<0.001
Risk score of 5-lncRNA-based classifier	1.047 (1.030, 1.065)	<0.001	1.040 (1.021, 1.059)	<0.001
Risk score of 4-miRNA-based classifier	1.038 (1.017, 1.059)	<0.001		
Age (≥55 vs. <55 years)	2.324 (1.276, 4.235)	0.006		
Gender (male vs. female)	1.563 (0.834, 2.928)	0.163		
Focus type (multifocal vs. unifocal)	1.143 (0.624, 2.094)	0.665		
**Pathologic stage (vs. stage I/II)**				
Stage III	2.510 (1.246, 5.053)	0.01		
Stage IV	3.842 (1.811, 8.153)	<0.001		
**Pathologic T stage (vs. T1/2)**				
T3	3.079 (1.584, 5.983)	<0.001	2.699 (1.374, 5.302)	0.004
T4	5.480 (2.100, 14.297)	<0.001	2.301 (0.726, 7.294)	0.157
Pathologic N stage (N1 vs. N0)	1.710 (0.928, 3.152)	0.086		
**Pathologic subtype (vs. classical papillary carcinoma)**				
Follicular papillary carcinoma	0.709 (0.276, 1.825)	0.476		
Tall cell papillary carcinoma	2.340 (1.028, 5.326)	0.043		

### Nomogram Analysis

**FIGURE 5:**
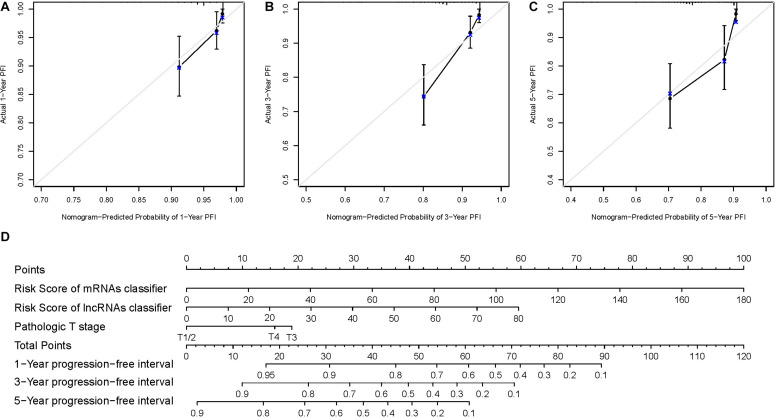
**(D)** The nomogram established with two RNA-based classifiers and pathologic T stage. **(A–C)** Calibration plots for the nomogram predicting 1-, 3-, and 5-year progression-free interval.

We constructed a nomogram for the prognostic prediction in patients with PTC based on the verified prognostic classifiers and clinical parameters (6-mRNA-based classifier, 5-lncRNA-based classifier, and pathologic T stage) in the multivariate Cox regression analysis. As shown in Figure 5D, the overall score could be calculated to estimate the PFI prognosis (1-, 3-, and 5-year PFI probabilities). The C-index of this nomogram model was 0.792 (95% CI: 0.716–0.867). The calibration curve demonstrated good discrimination of the nomogram model (Figures 5A–C). In general, this nomogram model could appropriately predict the PFI of PTC patients.

## Discussion

PTC is generally considered as an indolent carcinoma, and patients with PTC are commonly expected to have a favorable prognosis for disease-specific survival. However, the progression is not rare despite effective initial treatment (Mazzaferri and Jhiang, 1994; Grogan et al., 2013). Many stage systems have been developed to predict the risk of mortality in THCA patients. TNM stage system has been validated in both retrospective studies and prospective practice (Haugen et al., 2015). Nevertheless, molecular biomarkers were not integrated into the current stage system, and the predictive value for the progression of the PTC was not determined.

Increasing evidence has proved that the dysregulated ceRNA network which mainly involved lncRNA, miRNA, and mRNA was essential in many physiologic and pathological conditions including cancer initiation, progression, and metastasis (Karreth and Pandolfi, 2013). The lncRNA–miRNA–mRNA interactions have already been reported in many carcinomas, such as gastric cancer (Song et al., 2017), breast cancer (Lu et al., 2018), and hepatocellular cancer (Wang et al., 2019). As for PTC, although the role of ceRNA network has been investigated by some researchers (Liu H. et al., 2018; Zhuang et al., 2019), there are still some essentials to be explored. Firstly, the prognostic classifier for the progression of PTC should be built. Secondly, the internal validation of the classifier is indispensable to prove the stability of the classifier. Thirdly, the predictive value of the classifier should be compared with the existing stage system. Finally, a comprehensive prognostic model integrating multiple types of RNAs and clinical parameters could provide more accurate predictions.

In this study, based on the TCGA–THCA cohort, we constructed a PFI-related ceRNA network, established and validated three prognostic RNA-based classifiers for PFI, and built a nomogram including both the RNA signatures and clinical parameters.

There were some studies involving the ceRNA network of PTC (You et al., 2018; Zhao et al., 2018; Xu et al., 2019). However, only the global ceRNA network based on the differentially expressed lncRNAs, mRNAs, and miRNAs was constructed in these studies. Except the global ceRNA network, we also constructed a PFI-related ceRNA network that comprised five lncRNAs, four miRNAs, five mRNAs, and 14 edges. These RNAs and interactions might be valuable for further research.

Few studies have focused on the association between RNA-based classifiers and PTC progression. Chen et al. (2019) reported a prognostic lncRNA signature (TTTY10) that was identified through logistic regression model and incorporated into a nomogram to predict tumor recurrence risk of PTC based on the TCGA database. However, the recurrence data were essentially time-to-event data, and the logistic regression was not suitable to include both the event and time aspects as the outcome in the model. Moreover, as a clinical endpoint of TCGA-THCA data, PFI was considered more reliable than any other type of endpoints, such as overall survival, disease-specific survival, and disease-free interval (Liu J. et al., 2018). Therefore, in our study, we utilized the LASSO Cox regression method to select the prognostic RNA-based classifiers for predicting the PFI of PTC patients. Finally, a 6-mRNA-based classifier, a 5-lncRNA-based classifier, and a 4-miRNA-based classifier for PFI were constructed and validated. The results indicated that these classifiers could reasonably divide PTC patients into high- or low-risk groups with significant differences in PFI in the training set. The repeatability and practicability of the classifiers for the prediction of PFI were also verified in the testing set, indicating the potential prognostic value of the classifiers. Furthermore, we compared the discrimination ability of the classifiers with the TNM stage system. The ROC curve analysis showed that all the three classifiers had an obviously better predictive accuracy than the TNM stage system.

A comprehensive prognostic model integrating RNA-based classifiers and clinical parameters for PFI of the PTC patients was constructed based on univariate and multivariate Cox regression analyses. Only the 6-mRNA-based classifier, 5-lncRNA-based classifier, and the pathologic T stage were retained in the model as independent prognostic factors. A nomogram based on the prognostic model was established for the PFI prediction in patients with PTC. The C-index and calibration plot indicated good predictive accuracy of the nomogram.

Although our study revealed that the three RNA-based classifiers were related to the PFI of PTC, most of the RNAs in our classifiers have not been fully explored. Some of the RNAs have been reported in the previous study. PICALM interacting mitotic regulator (PIMREG) could promote breast cancer aggressiveness via sustaining nuclear factor (NF)-κB activation (Jiang et al., 2019). Progestin and adipoQ receptor family member (PAQR) 4 has been found to compete with S-phase kinase-associated protein (SKP) 2 for binding to the same region in cyclin-dependent kinase (CDK) 4, thereby abrogating SKP2-mediated ubiquitination of CDK4 and contributing to tumorigenesis (Wang et al., 2017). Growth arrest specific 2 like 2 (GAS2L2), also known as G2L2, was reported to be involved in mediating the cross talk between filamentous actin and microtubules (Stroud et al., 2014). Zhang et al. (2019) showed that LINC02154 was a risk factor for the prognosis of patients with laryngeal cancer. A study revealed that hsa-mir-486-1 may serve as a potential diagnostic biomarker of lung adenocarcinoma (Ren et al., 2019). Hsa-mir-6854 was reported as a potential prognostic miRNA biomarker for colon adenocarcinoma (Wei et al., 2018).

The present study also has some limitations. The study is based on the TCGA database. Further prospective clinical trials are needed to validate the results. Obtaining comprehensive clinical annotation was not the primary objective of the TCGA, thus, some important clinical factors such as treatment data were limited. The classifiers built in our study were based on the ceRNA theory; however, the specific molecular mechanisms of the RNAs in our classifiers are still unclear. The need for further studies is indisputable.

## Conclusion

In conclusion, a 6-mRNA-based classifier and a 5-lncRNA-based classifier were constructed and verified as novel and independent prognostic factors for PFI of PTC patients. The potential prognostic RNAs are worthy of further investigation prior to the utilization in the clinic.

## Data Availability Statement

Publicly available datasets were analyzed in this study. The data that support the findings of this study are openly available in TCGA at http://www.tcga.org/.

## Author Contributions

LiZ contributed to the conception and design. LuZ, SL, and CL contributed to the acquisition of the data. KW and JX contributed to the analysis and interpretation of the data, and manuscript writing and revising. All authors contributed to the article and approved the submitted version.

## Conflict of Interest

The authors declare that the research was conducted in the absence of any commercial or financial relationships that could be construed as a potential conflict of interest.

## Footnotes

1 https://cancergenome.nih.gov/

2 https://xena.ucsc.edu/

3 http://www.mircode.org/

4 http://www.targetscan.org/

5 http://mirtarbase.mbc.nctu.edu.tw/php/index.php

6 http://www.mirdb.org/

## Supplementary Material

FIGURE S1 Globe competing endogenous RNA network. The red indicates the upregulated RNAs, and the blue indicates the downregulated RNAs. The diamond represents lncRNAs, the round rectangle represents miRNAs, and the ellipse represents mRNAs.

FIGURE S2 **(A–F)** The risk score distribution in the training and testing sets. **(G–L)** The risk score distribution of progression status in the training and testing sets.
