# Supplementary material for: Prognostic lncRNA, miRNA, and mRNA Signatures in Papillary Thyroid Carcinoma
Source: Front Genet. 2020 Aug 4;11:805. doi: 10.3389/fgene.2020.00805 (PMC7417634; doi:10.3389/fgene.2020.00805)
Supplement: Supplementary file 1 [file Table_1.DOCX]

Table S1. The distribution of the clinical parameters in the training and testing sets.

| **Parameters** | **lncRNA** | | **miRNA** | | **mRNA** | |
| --- | --- | --- | --- | --- | --- | --- |
|  | Training set | Testing set | Training set | Testing set | Training set | Testing set |
| **Overall** | (n=248) | (n=245) | (n=249) | (n=248) | (n=248) | (n=245) |
| **Progression status** |  |  |  |  |  |  |
| Progression free | 220 (88.7%) | 222 (90.6%) | 0.129 (0.335) | 229 (92.3%) | 219 (88.3%) | 223 (91.0%) |
| Progression | 28 (11.3%) | 23 (9.4%) | 0.00 [0.00, 1.00] | 19 (7.7%) | 29 (11.7%) | 22 (9.0%) |
| **Age** |  |  |  |  |  |  |
| Mean (SD) | 48.3 (15.8) | 46.5 (16.0) | 47.4 (15.8) | 47.4 (15.9) | 47.1 (15.7) | 47.8 (16.0) |
| Median [Min, Max] | 47.0 [17.0, 89.0] | 46.0 [15.0, 85.0] | 46.0 [15.0, 89.0] | 47.0 [15.0, 88.0] | 46.0 [15.0, 88.0] | 48.0 [15.0, 89.0] |
| **Gender** |  |  |  |  |  |  |
| Female | 183 (73.8%) | 180 (73.5%) | 185 (74.3%) | 181 (73.0%) | 182 (73.4%) | 181 (73.9%) |
| Male | 65 (26.2%) | 65 (26.5%) | 64 (25.7%) | 67 (27.0%) | 66 (26.6%) | 64 (26.1%) |
| **Focus type** |  |  |  |  |  |  |
| Unknown | 3 (1.2%) | 7 (2.9%) | 3 (1.2%) | 7 (2.8%) | 1 (0.4%) | 9 (3.7%) |
| Multifocal | 115 (46.4%) | 105 (42.9%) | 108 (43.4%) | 113 (45.6%) | 110 (44.4%) | 110 (44.9%) |
| Unifocal | 130 (52.4%) | 133 (54.3%) | 138 (55.4%) | 128 (51.6%) | 137 (55.2%) | 126 (51.4%) |
| **Pathologic stage** |  |  |  |  |  |  |
| Unknown | 0 (0.0%) | 2 (0.8%) | 2 (0.8%) | 0 (0.0%) | 2 (0.8%) | 0 (0.0%) |
| Stage I | 137 (55.2%) | 138 (56.3%) | 138 (55.4%) | 140 (56.5%) | 143 (57.7%) | 132 (53.9%) |
| Stage II | 25 (10.1%) | 26 (10.6%) | 19 (7.6%) | 32 (12.9%) | 18 (7.3%) | 33 (13.5%) |
| Stage III | 58 (23.4%) | 53 (21.6%) | 61 (24.5%) | 51 (20.6%) | 57 (23.0%) | 54 (22.0%) |
| Stage IV | 28 (11.3%) | 26 (10.6%) | 29 (11.6%) | 25 (10.1%) | 28 (11.3%) | 26 (10.6%) |
| **Pathologic T stage** |  |  |  |  |  |  |
| T1 | 69 (27.8%) | 73 (29.8%) | 68 (27.3%) | 75 (30.2%) | 75 (30.2%) | 67 (27.3%) |
| T2 | 82 (33.1%) | 81 (33.1%) | 80 (32.1%) | 86 (34.7%) | 76 (30.6%) | 87 (35.5%) |
| T3 | 87 (35.1%) | 76 (31.0%) | 89 (35.7%) | 74 (29.8%) | 83 (33.5%) | 80 (32.7%) |
| T4 | 10 (4.0%) | 13 (5.3%) | 12 (4.8%) | 11 (4.4%) | 14 (5.6%) | 9 (3.7%) |
| TX | 0 (0.0%) | 2 (0.8%) | 0 (0.0%) | 2 (0.8%) | 0 (0.0%) | 2 (0.8%) |
| **Pathologic N stage** |  |  |  |  |  |  |
| N0 | 115 (46.4%) | 111 (45.3%) | 106 (42.6%) | 122 (49.2%) | 114 (46.0%) | 112 (45.7%) |
| N1 | 112 (45.2%) | 105 (42.9%) | 113 (45.4%) | 106 (42.7%) | 109 (44.0%) | 108 (44.1%) |
| NX | 21 (8.5%) | 29 (11.8%) | 30 (12.0%) | 20 (8.1%) | 25 (10.1%) | 25 (10.2%) |
| **Pathologic M stage** |  |  |  |  |  |  |
| Unknown | 0 (0.0%) | 1 (0.4%) | 1 (0.4%) | 0 (0.0%) | 1 (0.4%) | 0 (0.0%) |
| M0 | 136 (54.8%) | 141 (57.6%) | 137 (55.0%) | 140 (56.5%) | 142 (57.3%) | 135 (55.1%) |
| M1 | 4 (1.6%) | 5 (2.0%) | 6 (2.4%) | 3 (1.2%) | 4 (1.6%) | 5 (2.0%) |
| MX | 108 (43.5%) | 98 (40.0%) | 105 (42.2%) | 105 (42.3%) | 101 (40.7%) | 105 (42.9%) |
| **Pathologic subtype** |  |  |  |  |  |  |
| Classical | 175 (70.6%) | 181 (73.9%) | 178 (71.5%) | 180 (72.6%) | 183 (73.8%) | 173 (70.6%) |
| Follicular | 53 (21.4%) | 48 (19.6%) | 51 (20.5%) | 51 (20.6%) | 44 (17.7%) | 57 (23.3%) |
| Tall Cell | 20 (8.1%) | 16 (6.5%) | 20 (8.0%) | 17 (6.9%) | 21 (8.5%) | 15 (6.1%) |
