# Supplementary figures and images for: Prognostic lncRNA, miRNA, and mRNA Signatures in Papillary Thyroid Carcinoma
Source: Front Genet. 2020 Aug 4;11:805. doi: 10.3389/fgene.2020.00805 (PMC7417634; doi:10.3389/fgene.2020.00805)

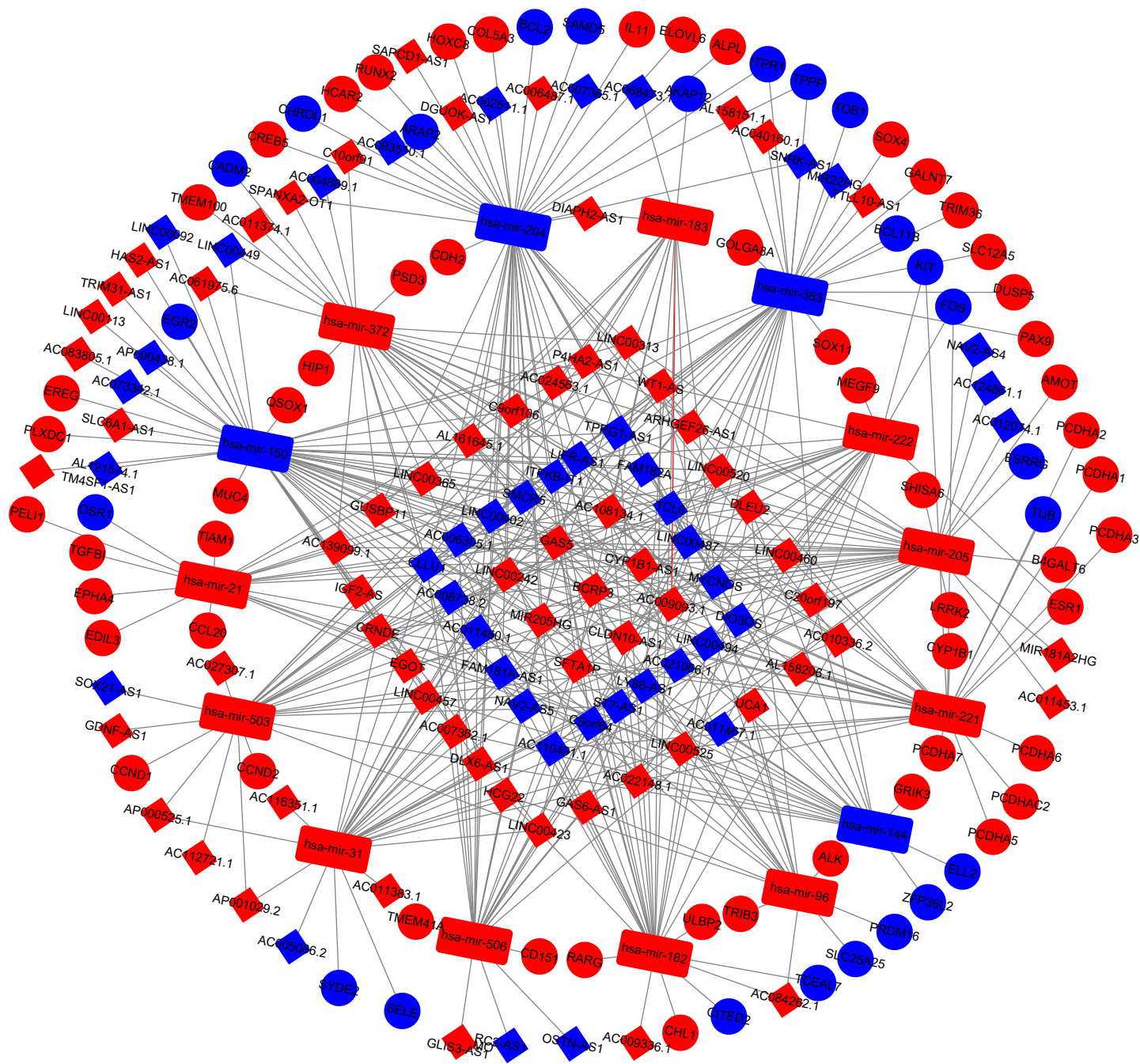

Supplement: FIGURE S1 — Globe competing endogenous RNA network. The red indicates the upregulated RNAs, and the blue indicates the downregulated RNAs. The diamond represents lncRNAs, the round rectangle represents miRNAs, and the ellipse represents mRNAs. [file Data_Sheet_1.PDF]
