# Supplementary material for: Prognostic lncRNA, miRNA, and mRNA Signatures in Papillary Thyroid Carcinoma
Source: Front Genet. 2020 Aug 4;11:805. doi: 10.3389/fgene.2020.00805 (PMC7417634; doi:10.3389/fgene.2020.00805)
Supplement: FIGURE S2 — (A–F) The risk score distribution in the training and testing sets. (G–L) The risk score distribution of progression status in the training and testing sets. [file Data_Sheet_2.PDF]

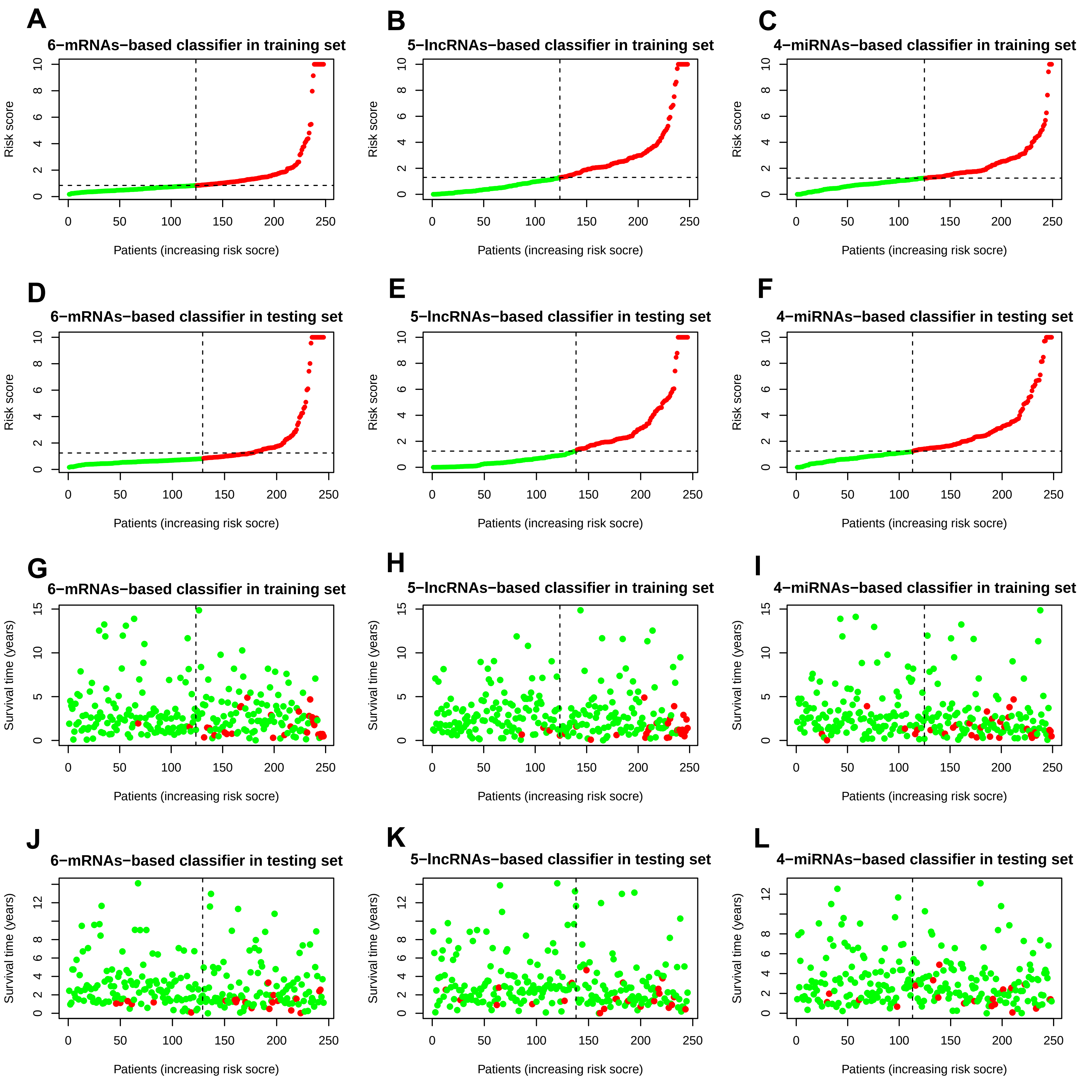

A
6−mRNAs−based classifier in training set
B
5−lncRNAs−based classifier in training set
C
4−miRNAs−based classifier in training set
D
6−mRNAs−based classifier in testing set
E
5−lncRNAs−based classifier in testing set
F
4−miRNAs−based classifier in testing set
G
6−mRNAs−based classifier in training set
H
5−lncRNAs−based classifier in training set
I
4−miRNAs−based classifier in training set
J
6−mRNAs−based classifier in testing set
K
5−lncRNAs−based classifier in testing set
L
4−miRNAs−based classifier in testing set
Risk score
Survival time (years)
Patients (increasing risk socre)
0
50
100
150
200
250
